# Fluorescence in situ hybridization reveals the evolutionary biology of minor clone of gain/amp(1q) in multiple myeloma

**DOI:** 10.1038/s41375-024-02237-3

**Published:** 2024-04-12

**Authors:** Jian Cui, Yuntong Liu, Rui Lv, Wenqiang Yan, Jingyu Xu, Lingna Li, Chenxing Du, Tengteng Yu, Shuaishuai Zhang, Shuhui Deng, Weiwei Sui, Mu Hao, Shuhua Yi, Dehui Zou, Lugui Qiu, Yan Xu, Gang An

**Affiliations:** 1 State Key Laboratory of Experimental Hematology, National Clinical Research Center for Blood Diseases, Haihe Laboratory of Cell Ecosystem, Institute of Hematology & Blood Diseases Hospital, Chinese Academy of Medical Science & Peking Union Medical College, Tianjin, 300020 China; 2 Tianjin Institutes of Health Science, Tianjin, 301600 China; 3 LeBow Institute for Myeloma Therapeutics and Jerome Lipper Center for Multiple Myeloma Center, Dana-Farber Cancer Institute, Harvard Medical School, Boston, MA USA

**Keywords:** Risk factors, Chromosome abnormality

## Abstract

Growing evidence suggests that gain or amplification [gain/amp(1q)] accumulates during disease progression of multiple myeloma (MM). Previous investigations have indicated that small gain/amp(1q) subclones present at the time of diagnosis may evolve into dominant clones upon MM relapse. However, the influence of a minor clone of gain/amp(1q) on MM survival, as well as the correlation between different clonal sizes of gain/amp(1q) and the chromosomal instability (CIN) of MM, remains poorly understood. In this study, we analyzed fluorescence in situ hybridization (FISH) results of 998 newly diagnosed MM (NDMM) patients. 513 patients were detected with gain/amp(1q) at diagnosis. Among these 513 patients, 55 had a minor clone (≤20%) of gain/amp(1q). Patients with a minor clone of gain/amp(1q) displayed similar survival outcomes compared to those without gain/amp(1q). Further analysis demonstrated patients with a minor clone of gain/amp(1q) exhibited a clonal architecture similar to those without gain/amp(1q). Lastly, our results showed a significant increase in the clonal size of the minor clone of gain/amp(1q), frequently observed in MM. These findings suggested that a minor clone of gain/amp(1q) might represent an earlier stage in the pathogenesis of gain/amp(1q) and propose a “two-step” process in the clonal size changes of gain/amp(1q) in MM.

## Introduction

Gain or amplification of 1q [gain/amp(1q)] serves as one of the most common secondary cytogenetic abnormalities (CAs) in multiple myeloma (MM) [1, 2]. Fluorescent in situ hybridization (FISH) reveals that gain/amp(1q) is detected in 30–40% of newly diagnosed MM (NDMM) cases at different cutoff values [3]. Furthermore, when patients with MM experience relapses, a higher percentage of them are reported to have gain/amp(1q) [4–8]. The progressive accumulation of gain/amp(1q) during disease progression suggests its involvement in MM’s resistance to treatment [9]. Additionally, a growing body of evidence indicates that patients with gain/amp(1q) have shorter survival rates compared to those without this abnormality [3, 10–12]. Consequently, gain/amp(1q) has been incorporated into several prognostic staging systems, including the second revision of the International Staging System (R2-ISS) and the Mayo Additive Staging System (MASS) [13, 14], as one of the high-risk cytogenetic factors.

Gain/amp(1q) is not solely a result of chromosomal instability (CIN) in MM but is also attributed to jumping translocations of 1q12, contributing to the chromosomal instability (CIN) phenotype in MM. This leads to unbalanced aberrations in the receptor chromosomes and other secondary CAs, including deletion of 17p [del(17p)] and translocation of MYC [15, 16]. A recent single-cell study has discovered that detectable gain/amp(1q) at relapse in MM may initially present as a minor clone at diagnosis [6]. The survival rates of patients with newly acquired gain/amp(1q) at relapse are similar to those with gain/amp(1q) at the time of diagnosis [6, 7]. Moreover, evidence suggests that MM subclones with gain/amp(1q) frequently expand during different treatments [9], implying that a minor clone of gain/amp(1q) may be associated with CIN in MM. Nevertheless, most centers have now established a cutoff value of 20% [4, 8, 12, 17–19] or 30% [20] for gain/amp(1q), with only a few using slightly smaller cutoff values of 3.5% [10], 5% [21] or 5.5% [22] for gain/amp(1q). These relatively large cutoff values have made it unclear whether a minor clone of gain/amp(1q) should be considered a high-risk CA in MM and whether it is associated with CIN in MM.

To address these questions, we conducted an analysis of the genetic profiles of 998 patients with NDMM. Our goal was to assess the impact of gain/amp(1q) at various clonal sizes and the copy number of 1q. We then investigated the correlation between gain/amp(1q) and other CAs, aiming to uncover the relationship between gain/amp(1q) and CIN in MM. Additionally, we explored the prognostic significance of having a minor clone of gain/amp(1q) in conjunction with other CAs. Lastly, we delved into the clonal evolution of the minor clone of gain/amp(1q) by analyzing paired FISH results from 13 patients with MM at the time of diagnosis and their first relapse.

## Materials and methods

### Patients

The patients included in this study were drawn from the MM database of the National Longitudinal Cohort of Hematological Diseases (NICHE, NCT04645199). Inclusion criteria required patients to meet the International Myeloma Working Group (IMWG) consensus definition of MM [23] and to have the required FISH data, including testing for gain/amp(1q), del(1p), del(17p), del(13q), and IgH translocation. This study encompassed MM patients diagnosed between January 2014 and June 2021. A total of 998 NDMM patients were identified. All patients provided informed consent in accordance with the Declaration of Helsinki. The study received approval from the local institutional ethics committees of the Institute of Hematology and Blood Diseases Hospital, Chinese Academy of Medical Science & Peking Union Medical College (Certificate: IIT2020023-EC-1).

### FISH testing

The iFISH (interphase fluorescence in situ hybridization) technique used in this study has been previously documented [4, 5, 24]. Bone marrow (BM) aspirate samples, anticoagulated with EDTA, were collected, and CD138^+^ plasma cells (PCs) were enriched using CD138^+^ magnetic beads (Miltenyi Biotec, Paris, France), enabling a post-sorting purity higher than 90% as previously described [25, 26]. CAs were then analyzed on the purified PCs, and a total of 200 interphase nuclei were tested. DNA probes specific for 13q14, 17p13, and IgH dual color break-apart rearrangement probe were purchased from Abbott Molecular/Vysis (Des Plaines, IL, USA). If an IgH rearrangement was found by the IgH break-apart probe, reflex testing was performed using dual-color, dual-fusion probes for t(4;14)(p16;q32), t(11;14)(q13;q32), t(14;16)(q32;q23) and t(14;20)(q32;q12) to identify the translocation partner (Abbott Molecular/Vysis). And the Cytocell dual-color CKS1B/CDKN2C probe (Oxford Gene Technology) was used for detecting gain/amp(1q) and del(1p). According to our previous ROC analyses [5], the cutoff values for del(17p), gain/amp(1q) and del(13q) were set at 50%, 20% and 10%, respectively. For del(1p) and IgH translocations, the positivity threshold was 10%. Furthermore, the mean + 3 standard deviations of gain/amp(1q) calculated from the normal control is 2.12% in our center, and this value is set as our in-house established technical cutoff. A minor clone of gain/amp(1q) or gain/amp(1q) ≤ 20% is defined as the detection of gain/amp(1q) in purified PCs exceeding 2.12% but not exceeding 20%. In accordance with the Revised International Staging System (R-ISS) [27], patients with del(17p), t(4;14), or t(14;16) were classified as having high-risk CAs, while those without these CAs were categorized as standard-risk.

### Statistical analysis

The objective of this study was to explore the connection between CAs and survival outcomes in MM patients. We defined progression-free survival (PFS) as the duration from diagnosis to the date of death, initial progression, or the last follow-up. Overall survival (OS) was calculated from the time of diagnosis to the date of death or the last follow-up. Hazard ratios (HR) and 95% confidence intervals (CI) were determined using the Cox regression model. Continuous variables were compared using either the Student’s *t* test or the Mann–Whitney U test, depending on the distribution of the variables. The χ2 test or Fisher’s exact test was employed to evaluate the statistical significance of categorical variables among different groups. A two-sided *p* value of less than 0.05 was regarded as statistically significant. All statistical analyses were conducted using SPSS (version 26.0; IBM, Chicago, IL, USA) and R (version 4.2.0; R Foundation, Vienna, Austria).

## Results

### The prognostic significance of gain/amp(1q) at different clonal sizes in NDMM

In this study, a total of 998 consecutive patients who received care at the Blood Diseases Hospital of the Chinese Academy of Medical Sciences and had cytogenetic data examined by FISH at the time of diagnosis (testing for gain/amp(1q), del(13q), del(17p), del(1p), and IgH translocation) were included (Fig. S1). The median follow-up time for the entire cohort was 38.2 months. The baseline characteristics of all patients are detailed in Table 1. The median age was 60 years, with 48.7% and 23.2% of patients classified as International Staging System (ISS) stage III and R-ISS stage III, respectively. Del(13q), del(17p), and del(1p) were observed in 47.8%, 6.5%, and 5.1% of patients in our cohort, respectively. Moreover, more than half of the patients at diagnosis were found to have IgH translocation (578/998, 57.9%) (Table 1).

**Table 1:** Baseline characteristics.

Characteristic	N = 998
Age, years, median (IQR)	60 (51–65)
Male	557 (56)
Paraprotein type, n (%)	
IgG	460 (46.1)
IgA	253 (25.4)
IgD	50 (5.0)
Biclonal	1 (0.1)
Light chain only	173 (17.3)
Missing	61 (6.1)
Light chain type, n (%)	
Lambda	487 (48.8)
Kappa	445 (44.6)
Missing	66 (6.6)
ISS stage, n (%)	
1	165/924 (17.9)
2	309/924 (33.4)
3	450/924 (48.7)
R-ISS stage, n (%)	
1	109/831 (13.1)
2	529/831 (63.7)
3	193/831 (23.2)
Hb, g/dL, median (IQR)	9.9 (8.1–12.1)
Platelets, ×10^9^/L, median (IQR)	188 (129–244)
Serum creatinine, umol/L, median (IQR)	81 (65–117)
LDH, units/L, median (IQR)	169 (137–220)
B2M, ug/mL, median (IQR)	4.7 (3.0–8.6)
High-risk cytogenetic, n (%)	
Any HRCA^a^ (n = 840)	233 (27.7)
With at least one CA^b^	826 (82.8)
IgH translocation	578 (57.9)
t(4;14) (n = 839)	147 (17.5)
t(11;14) (n = 831)	132 (15.9)
t(14;16) (n = 836)	27 (3.2)
t(14;20) (n = 809)	3 (0.3)
t(14;undefined)^c^ (n = 828)	99 (12.0)
del(17p)	65 (6.5)
del(13q)	477 (47.8)
del(1p)	51 (5.1)

*IQR* interquartile range, *ISS* International Staging System, *R-ISS* Revised International Staging System, *Hb* hemoglobin, *LDH* lactate dehydrogenase, *B2M* β2-microglobulin, *HRCA* high-risk chromosome abnormality.

^a^High-risk CA: presence of t (4;14), t(14;16), and/or del(17p).

^b^The cutoff value for del(17p), gain/amp(1q), del(13q), del(1p) and IgH translocations were set at 50%, 20%, 10%, 10% and 10%, respectively.

^c^t(14; undefined): patients with an undefined abnormality of the 14q32 locus that did not correspond to one of the above three described common translocations.

**Fig. 1:**
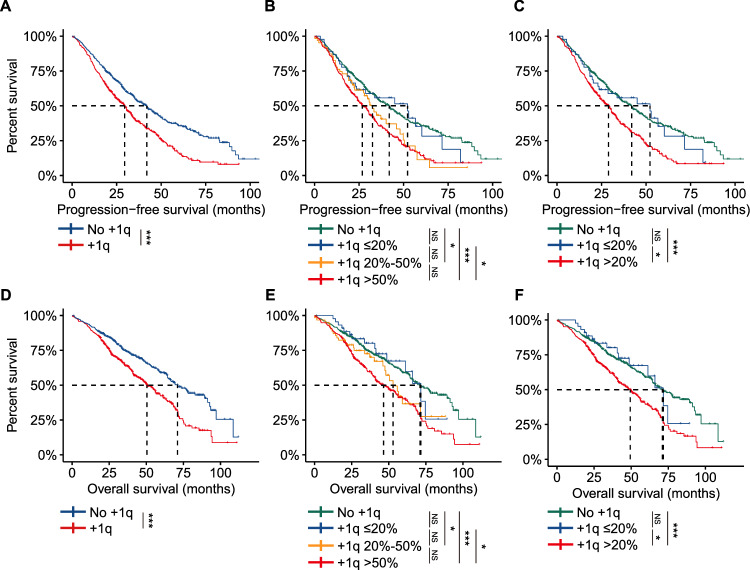
The prognostic significance of gain/amp(1q) at different clonal sizes in NDMM. Kaplan-Meier analysis of PFS (**A**) and OS (**D**) by gain/amp(1q). Kaplan-Meier analysis of PFS (**B**) and OS (**E**) by clonal size. NDMM patients with gain/amp(1q) are grouped using cutoff values of 20% and 50%. Kaplan–Meier analysis of PFS (**C**) and OS (**F**) by clonal size. NDMM patients with gain/amp(1q) are grouped using a cutoff value of 20%. NS not significant, *P < 0.05, ***P < 0.001, by two-sided log-rank test.

Since the cutoff value of gain/amp(1q) is different among different centers (Table S1). We thus did not set a specific cutoff value for gain/amp(1q) in this study to investigate the prognostic significance of gain/amp(1q) at different clonal sizes. Gain/amp(1q) was detected in 513 patients at the time of diagnosis. Furthermore, significantly shorter PFS and OS were observed in patients with gain/amp(1q) compared to those without gain/amp(1q) (PFS: 29.5 months vs. 41.9 months, HR = 1.58, 95% CI: 1.33–1.89, P < 0.001; OS: 50.4 months vs. 71.0 months, HR = 1.67, 95% CI: 1.34–2.07, P < 0.001) (Fig. 1A, D).

Based on the proportion of PCs involved, patients with gain/amp(1q) were subsequently categorized into four groups: no gain/amp(1q), gain/amp(1q) ≤ 20%, gain/amp(1q) 20%-50%, and gain/amp(1q) > 50%. The median PFS was 41.9 months, 52.2 months, 32.5 months, and 26.8 months, respectively, while the median OS was 71.0 months, 71.6 months, 52.9 months, and 46.5 months for these four subgroups (Fig. 1B, E). Our results showed that patients with gain/amp(1q) ≤ 20% had similar survival to those without gain/amp(1q), while patients with gain/amp(1q) in less than 50% of clonal PCs (20–50%) and gain/amp(1q) in more than 50% of clonal PCs experienced similar survival. Further investigation revealed that patients with gain/amp(1q) ≤ 20% experienced significantly better survival compared to those with gain/amp(1q) > 20% (Fig. S2A, S2B). Thus, based on the survival curves, 20% was then selected as the cutoff value to divide the patients into three subgroups: no gain/amp(1q), gain/amp(1q) ≤ 20%, and gain/amp(1q) > 20%. The median PFS was 28.9 months, and the median OS was 49.4 months for patients with gain/amp(1q) > 20% (Fig. 1C, F).

In line with prior studies (Table S2) [10, 12, 18, 21, 26], including our own investigation [4, 28], our findings indicated that gaining more than one copy of 1q did not confer additional prognostic significance to gain/amp(1q). There was no significant difference in median PFS and median OS between patients with three copies and those with four or more copies of 1q (PFS: 29.6 months vs. 29.5 months, P = 0.640; OS: 50.4 months vs. 50.0 months, P = 0.963) (Fig. S3A, S3B). However, for patients with gain/amp(1q) ≤ 20%, having four or more copies of 1q was associated with a significantly shorter PFS compared to having three copies of 1q (56.7 months vs. 19.8 months, P = 0.029) (Fig. S3C). Moreover, for patients with gain/amp(1q) ≤ 20%, our results indicated no significant difference in OS between patients with three copies or four or more copies of 1q (Fig. S3D). Patients with at least four copies of 1q also exhibited comparable PFS and OS compared to those with three copies of 1q, either for gain/amp(1q) 20%-50% or gain/amp(1q) 50% (Fig. S3E–3H). Furthermore, no statistically significance was observed between different clonal sizes and copy numbers in patients with gain/amp(1q) in the aspect of both PFS and OS (Fig. S4A, S4B).

### Copy number and clonal size evolution of gain/amp(1q) and its relationship with CIN phenotype in MM

**Fig. 2:**
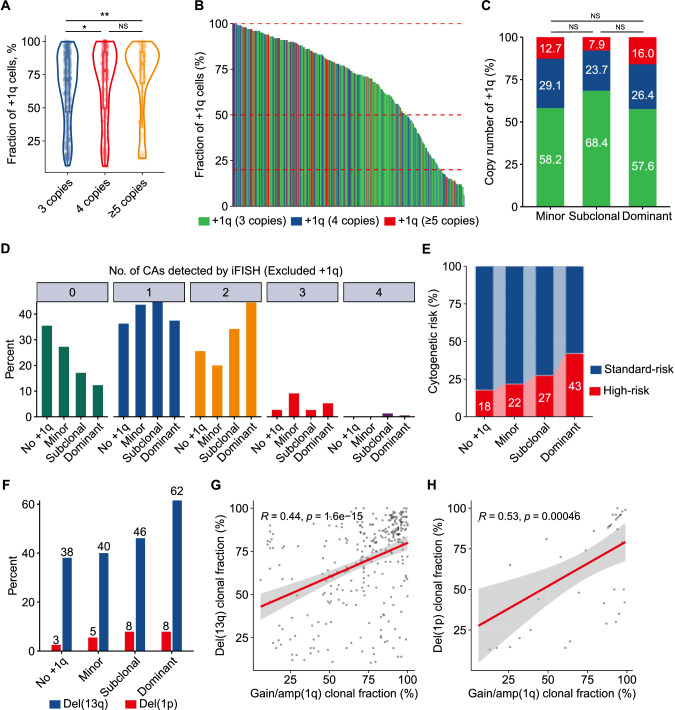
Copy number and clonal size evolution of gain/amp(1q) and its relationship with CIN phenotype in MM. **A** The proportion of cells with gain/amp(1q) is indicated by the height of the bar on the y-axis. The proportion of cells with three, four, or ≥five copies of 1q21 in each sample is indicated by green, blue, and red, respectively. A total of 513 NDMM with gain/amp(1q) are ordered from the lowest to the highest proportion of cells with gain/amp(1q) from right to left on the x-axis. **B** Volin plot of the cell fraction of gain/amp(1q) in patients detected with different copy numbers of 1q. NS, not significant, *P < 0.05, **P < 0.01, by two-sided unpaired Student’s *t* tests. **C** Bar plot comparing the proportions of distribution of patients detected with different copy numbers of 1q according to the clonal size of gain/amp(1q). NS, not significant, ***P < 0.001, by 2-sided χ² test. **D** Frequency bar plot showing the number of CAs other than gain/amp(1q) detected by FISH among NDMM patients without gain/amp(1q) or with different clonal sizes of gain/amp(1q). **E** Comparison of proportion of high-risk CAs among patients without gain/amp(1q) or with different clonal sizes of gain/amp(1q). **F** Percentage frequency of genetic changes associated with minor, subclonal, and clonal gain/amp(1q). **G** Scatter plots demonstrate the relationship of gain/amp(1q) clonal fraction and del(13q) clonal fraction for patients with concomitant gain/amp(1q) and del(13q). **H** Scatter plots demonstrate the relationship of gain/amp(1q) clonal fraction and del(1p) clonal fraction for patients with concomitant gain/amp(1q) and del(1p).

To provide further insights into the characteristics of clonal size and copy number of gain/amp(1q) and their relationship, we initially grouped the patients into three categories based on the copy number of 1q: three copies, four copies, and five or more copies. The median clonal sizes of gain/amp(1q) for these groups were 71%, 78%, and 83.5%, respectively. Notably, significantly higher clonal sizes were observed in patients with five or more copies or four copies of 1q compared to those with three copies of 1q (Fig. 2A). Subsequently, we applied cutoff values of 20% and 50% to categorize the clonal size of gain/amp(1q) into minor (≤20%), subclonal (20–50%), and dominant (>50%) clones. In our cohort, 55, 76, and 382 NDMM patients were identified with a minor, subclonal, and dominant clone of gain/amp(1q), respectively (Fig. 2B).

Our results showed that patients received similar treatment regimens. For patients without gain/amp(1q), with a minor, subclonal, and dominant clone of gain/amp(1q), PI-containing regimens were received in 75%, 81%, 82% and 73% of them, respectively (P = 0.173). Furthermore, there was also no significant difference in the proportion of patients in these four groups who received first-line autologous hematopoietic stem cell transplantation (dominant: 34%; subclonal: 42%; minor: 26%; no gain/amp(1q): 34%, P = 0.416) (Table S3).

Interestingly, our results showed similar copy number architecture between patients with a minor clone and those with a dominant or subclonal clone of gain/amp(1q) (Fig. 2C). Further analyses revealed that progressively higher numbers of CAs were detected in patients without gain/amp(1q) and in those with a minor, subclonal, and dominant clone of gain/amp(1q). For patients without gain/amp(1q), with a minor, subclonal, and dominant clone of gain/amp(1q), at least one CA was detected in 65%, 73%, 83%, and 88% of them, respectively (Fig. 2D). Moreover, for patients with no gain/amp(1q) and with a minor clone of gain/amp(1q), 18% and 22%, respectively, had at least one high-risk CA, while for patients with a subclonal and a dominant clone of gain/amp(1q), 27% and 43% of patients were detected with at least one high-risk CA at the time of diagnosis, respectively (Fig. 2E).

Despite being a rare high-risk cytogenetic event at diagnosis, more cases of del(17p) were observed in patients with a dominant clone of gain/amp(1q) (Fig. S5A). Our results also showed that patients with concomitant gain/amp(1q) and del(17p) had slightly higher copy numbers of 1q compared to those with only gain/amp(1q) (Fig. S5B). For standard-risk CAs such as del(13q) and del(1p), a dominant or subclonal clone, compared to a minor clone or no gain/amp(1q), was associated with higher rates of del(13q) (dominant: 62%; subclonal: 46%; minor: 40%; no gain/amp(1q): 38%) and del(1p) (dominant: 8%; subclonal: 8%; minor: 5%; no gain/amp(1q): 3%) (Fig. 2F).

Additionally, a significant correlation of clonal size was observed for patients with concomitant del(13q) and gain/amp(1q) (R = 0.44, P < 0.001) (Fig. 2G). A similar correlation was likewise observed for patients with both del(1p) and gain/amp(1q) (R = 0.53, P < 0.001) (Fig. 2H). Finally, regarding clinical characteristics such as the ISS stage, the rates of ISS stage III were increased in patients with a dominant or subclonal clone of gain/amp(1q) compared to those with a minor clone of gain/amp(1q) or no gain/amp(1q) (Fig. S5C). In summary, the associations between gain/amp(1q) clonal size and increasing rates of other secondary CAs suggested that gain/amp(1q) was related to the CIN phenotype in MM.

### Concomitant del(1p) and minor clone of gain/amp(1q) are associated with a poor clinical outcome in MM

**Fig. 3:**
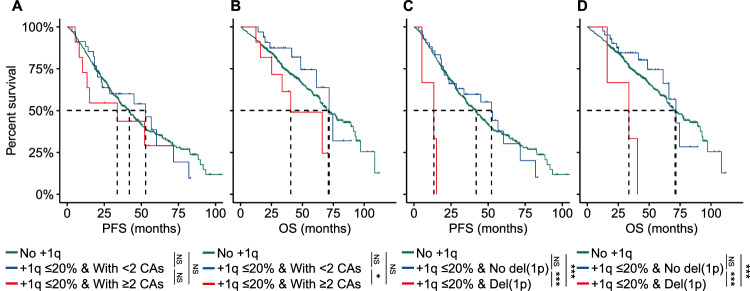
Concomitant del(1p) and minor clone of gain/amp(1q) are associated with a poor clinical outcome in MM. **A**, **B** Kaplan-Meier analysis of PFS (**C**) and OS (**D**) by gain/amp(1q) and number of CAs detected by FISH. Kaplan-Meier analysis of PFS (**C**) and OS (**D**) by gain/amp(1q) and del(1p) detected by FISH. NS, not significant, *P < 0.05, **P < 0.01, ***P < 0.001, by two-sided log-rank test.

Previous studies have demonstrated that jumping translocations of 1q can lead not only to the amplification of 1q but also to other secondary CAs in MM, including MYC translocations and del(16q) [15, 16]. We hypothesized that there might be a subset of patients with a minor clone of gain/amp(1q) who had worse survival due to concomitant high-risk genetic factors. To investigate this, we initially compared the survival outcomes of patients with both a minor clone of gain/amp(1q) and at least two CAs (other than gain/amp(1q)) to those with a minor clone of gain/amp(1q) and fewer than two CAs. Despite similar PFS between these two groups (+1q ≤ 20% & <2 CAs: 52.9 months vs. +1q ≤ 20% & ≥2 CAs: 33.7 months, P = 0.370) (Fig. 3A), patients with +1q ≤ 20% & ≥2 CAs experienced significantly shorter OS than those with +1q ≤ 20% & <2 CAs (+1q ≤ 20% & <2 CAs: 71.6 months vs. +1q ≤ 20% & ≥2 CAs: 40.6 months, P = 0.035) (Fig. 3B).

Further analysis revealed significantly shorter PFS and OS in patients with concomitant del(1p) and a minor clone of gain/amp(1q) compared to those with a minor clone of gain/amp(1q) and without del(1p) (PFS: +1q ≤ 20% & no del(1p): 52.2 months vs. +1q ≤ 20% & del(1p): 13.4 months, P < 0.001; OS: +1q ≤ 20% & no del(1p): 71.6 months vs. +1q ≤ 20% & del(1p): 33.6 months, P < 0.001) (Fig. 3C, D). In conclusion, our results suggested that although a minor clone of gain/amp(1q) was not inherently associated with a poor prognosis in MM, the coexistence of more than two CAs in a subset of patients with a minor clone of gain/amp(1q) might lead to a poor prognosis in this specific group of patients.

### Clonal evolution of minor clone of gain/amp(1q)

**Fig. 4:**
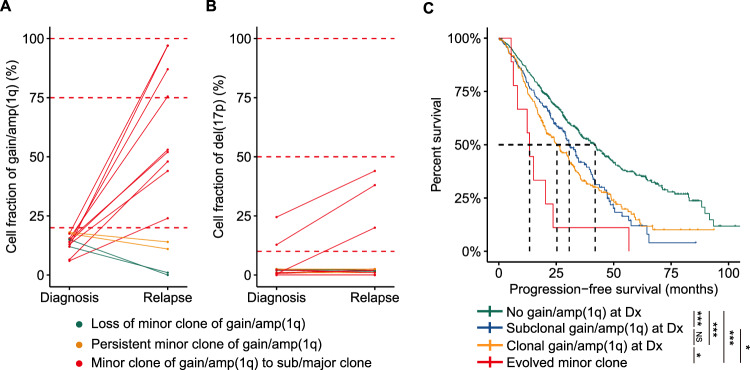
Clonal evolution of minor clone of gain/amp(1q). **A** The change in cell fraction of gain/amp(1q) between two time points. Different colors demonstrate three different evolutionary patterns of gain/amp(1q) between diagnosis and relapse. **B** The change in cell fraction of del(17p) between two time points. Different colors demonstrate three different evolutionary patterns of gain/amp(1q) between diagnosis and relapse. **C** Kaplan-Meier analysis of PFS by different gain/amp(1q) clonal sizes at diagnosis and evolved minor clone between two time points. NS not significant, *P < 0.05, **P < 0.01, ***P < 0.001, by two-sided log-rank test.

Given recent findings that a minor clone of gain/amp(1q) at diagnosis can become a major clone at relapse in MM [6], and considering previous studies that have suggested a significantly higher proportion of patients carrying gain/amp(1q) at relapse compared to diagnosis [5, 8], we delved into the clonal evolution of the minor clone of gain/amp(1q) by conducting longitudinal FISH examinations at diagnosis and relapse. In our cohort, 13 patients with a minor clone of gain/amp(1q) at diagnosis underwent FISH testing at their first relapse (Table S4, S5). Our results indicated that nine cases (69%) saw the evolution of the minor clone at diagnosis into a subclonal/dominant clone at first relapse, while two patients (15%) maintained a minor clone of gain/amp(1q) both at diagnosis and relapse. Only two patients (15%) experienced the loss of the minor clone of gain/amp(1q) at relapse (Fig. 4A). Further analysis revealed that the clonal evolution of the minor clone of gain/amp(1q) was accompanied by an expanded clonal size of del(17) or by newly acquired del(17p) (Fig. 4B). Finally, patients with a significant increase in clonal size of gain/amp(1q) had significantly shorter PFS than patients with a subclonal and a dominant clone of del(1q) at diagnosis (Fig. 4C), while no significant differences were observed for OS among these groups (Fig. S6A). Thus, our study suggested that a minor clone of gain/amp(1q) was prone to evolve into a dominant clone at relapse and was also correlated with the clonal evolution of del(17p).

## Discussion

In this study, we conducted a retrospective analysis of 998 MM patients who had the necessary cytogenetic profiles at diagnosis. Our research focused on several key aspects, including the prognostic significance of a minor clone of gain/amp(1q), the correlation between different clonal sizes of gain/amp(1q) and CIN, the co-occurrence of a minor clone of gain/amp(1q) with other cytogenetic factors and its prognostic value, and the pattern of clonal evolution of a minor clone of gain/amp(1q). In our cohort, out of the 513 patients with gain/amp(1q) at diagnosis, 55 (10.7%) had a minor clone of gain/amp(1q).

Regarding the cutoff values for gain/amp(1q), many centers, including ours, tend to use relatively larger values, such as 20% [4, 8, 12, 17–19] or 30% [20]. However, some centers, like the Mayo Clinic, employ a smaller cutoff value of 3.5% for gain/amp(1q) [10]. Previous studies have shown that patients with a minor clone of gain/amp(1q) (10–20%) have similar survival compared to those without gain/amp(1q) or with gain/amp(1q) < 10% [5]. Furthermore, several studies have reported that PFS and OS are similar between patients with gain/amp(1q) in less than 50% of clonal PCs and those with gain/amp(1q) in more than 50% of clonal PCs [4, 12, 29]. In our study, patients with a minor clone of gain/amp(1q) experienced similar survival compared to those without gain/amp(1q). Additionally, the PFS and OS curves were similar between patients with gain/amp(1q) in less than 50% of clonal PCs (20–50%) and those with gain/amp(1q) in more than 50% of clonal PCs. Thus, the importance of gain/amp(1q) as well as the size of the subpopulation affected might reflect the disease evolution as well as resistance to the treatment used in MM.

The prognostic significance of the copy number of 1q is still under investigation. Some studies have suggested that copy number variation does not provide additional prognostic value [4, 12, 18, 21, 26, 30, 31], while others have indicated that amp(1q) is associated with shorter survival compared to gain(1q) [11, 32]. Our results also suggested that patients with gain(1q) had similar survival to those with amp(1q). However, we observed that patients with a minor clone of amp(1q) had significantly shorter PFS compared to those with a minor clone of gain(1q). Thus, our findings supported the use of a 20% cutoff value for gain/amp(1q). While patients with a minor clone of gain/amp(1q) exhibited similar survival compared to those without gain/amp(1q), a minor clone of amp(1q) was associated with a negative impact on the prognosis of MM.

Gain/amp(1q) is one of the most common CAs in patients with MM and is associated with CIN [16, 33]. Research has shown that jumping translocations involving the whole or part of the long arm of chromosome 1 can lead to the gain of 1q in MM [15, 16, 34, 35]. Moreover, as a secondary CA in MM, previous genomic studies have indicated that the occurrence of gain/amp(1q) typically takes place at a relatively early stage in the pathogenesis of MM [36, 37]. In our present study, we observed that patients with a subclonal and dominant clone of gain/amp(1q) displayed markers of high-risk MM, including higher rates of CAs, del(13q), and/or del(1p). In contrast, patients with a minor clone of gain/amp(1q) exhibited a similar clonal architecture to those without gain/amp(1q). This finding strongly suggested a “two-step” process in the changes in clonal size of gain/amp(1q) in MM. These data further reinforced the concept that the evolution of clonal size in gain/amp(1q) is closely linked to CIN in MM. Moreover, this observation was consistent with previous studies that have indicated the expansion of genetically abnormal PCs as the disease progresses in MM [38]. Although there is currently a lack of experimental evidence to conclusively support the “two-step” pathogenesis of gain/amp(1q), it is worth noting that significantly higher numbers of tumor-associated macrophages and inflammatory classical dendritic cells are found in the tumor microenvironment of patients with a subclonal/dominant clone of gain/amp(1q) compared to those without gain/amp(1q) or with a minor clone of gain/amp(1q) [9].

While there is substantial evidence indicating that the presence of gain/amp(1q) is associated with shorter survival in MM patients [3, 39], there are also studies that demonstrate the co-occurrence of gain/amp(1q) with other clinically and cytogenetically high-risk factors, identifying a subgroup of ultra-high risk patients [19, 40]. In a study conducted by the Myeloma Genome Project, comprehensive genomic data are collected from 1,273 NDMM patients. Their results suggest that patients with both gain/amp(1q) and ISS stage III have dismal survival outcomes, similar to those with a “double-hit” involving del(17p) and TP53 mutation [40]. In our present study, our primary focus was on assessing the prognostic value of the concurrent presence of a minor clone of gain/amp(1q) and other high-risk cytogenetic factors. We defined a “double-hit” high-risk subgroup as the co-occurrence of a minor clone of gain/amp(1q) and del(1p), comprising 5.5% (3 out of 55) of the population. In line with a previous study [19], the presence of both a minor clone of gain/amp(1q) and del(1p) was associated with a poor survival outcome in MM. This finding suggested that while a minor clone of gain/amp(1q) alone might be considered a standard-risk CA in MM, the combination of a minor clone of gain/amp(1q) and del(1p) identified a subset of patients with a poor prognosis.

The acquisition of gain/amp(1q) during the follow-up is not uncommon in MM. As recently reported in our study of 188 MM patients who have paired FISH results at the time of diagnosis and first relapse [41], we have found that 18.6% (35 out of 188) of patients acquire gain/amp(1q) at their first relapse. Furthermore, an increase in the copy number is observed in 21 patients. In another study involving 43 MM patients who undergo paired targeted sequencing at diagnosis and first relapse, newly acquired gain/amp(1q) is observed in eight patients (18.6%) [42]. In a more recent study of 956 patients who are tested for CAs by FISH at diagnosis and first relapse, newly acquired gain/amp(1q) is observed in 4.5% (43 out of 956) of patients [6]. Within our cohort, 13 patients with a minor clone of gain/amp(1q) had paired FISH results at their first relapse. Our results indicated that nine cases (69%) experienced the evolution of the minor clone at diagnosis into a subclonal or dominant clone at the first relapse.

This study had its limitations due to its retrospective design. Despite the large size of our cohort, we only had 55 patients with a minor clone of gain/amp(1q) in our study. Additionally, only 13 patients in our cohort had paired FISH results at both the time of diagnosis and at relapse, as some patients with a minor clone of gain/amp(1q) had either not yet experienced a relapse or did not undergo FISH examination at the time of relapse. Since a previous study by Jones et al. [43] suggested that the use of the use of maintenance therapy and the depth of response can impact the evolutionary patterns seen at relapse, the interpretation of the results of our study needs to take into account the depth of response and differences in treatment. Furthermore, further experimental studies are necessary to confirm whether the pathogenesis of gain/amp(1q) indeed follows the “two-step” process as we assumed. Finally, since the technical cutoff value of 2.12% was obtained from bone marrow mononuclear cells rather than plasma cells of healthy controls, it is primarily derived from other cellular components of the bone marrow. This may inevitably introduce false-positive detection results. Therefore, we believe that in the future, it is necessary to employ more sensitive detection methods, such as single-cell DNA sequencing, to further investigate our findings.

In conclusion, our study showed that for MM patients with gain/amp(1q) at the time of diagnosis, approximately 10% of these cases involved a minor clone. There was considerable variation in survival outcomes among patients with a minor clone of gain/amp(1q), and the co-occurrence of two or more CAs, other than gain/amp(1q), or concurrent del(1p), was associated with a poorer prognosis in MM. Additionally, our investigation into the clonal evolution of the minor clone of gain/amp(1q) revealed that a substantial increase in the clonal size of the minor clone of gain/amp(1q) was not an uncommon occurrence in MM and was correlated with a significant decrease in patients’ survival outcomes.

### Supplementary information

Figure S1-S6, Table S1-S5.

## Data Availability

The datasets generated and/or analyzed during the current study are available from the corresponding author at angang@ihcams.ac.cn upon reasonable request. All other relevant data supporting the key findings of this study are available within the article and its Supplementary files or from the corresponding author at angang@ihcams.ac.cn upon reasonable request.
