# Supplementary material for: Fluorescence in situ hybridization reveals the evolutionary biology of minor clone of gain/amp(1q) in multiple myeloma
Source: Leukemia. 2024 Apr 12;38(6):1299–306. doi: 10.1038/s41375-024-02237-3 (PMC11147758; doi:10.1038/s41375-024-02237-3)
Supplement: Supplementary file 1 — Figure S1-S6, Table S1-S5. [file 41375_2024_2237_MOESM1_ESM.docx]

**Supplementary data**

**Fluorescence in situ hybridization reveals the evolutionary biology of minor clone of gain/amp(1q) in multiple myeloma**

Jian Cui^1,2^, Yuntong Liu^1,2^, Rui Lv^1,2^, Wenqiang Yan^1,2^, Jingyu Xu^1,2^, Lingna Li^1,2^, Chenxing Du^1,2^, Tengteng Yu^1,2^, Shuaishuai Zhang^1,2^, Shuhui Deng^1,2,3^, Weiwei Sui^1,2^, Mu Hao^1,2^, Shuhua Yi^1,2^, Dehui Zou^1,2^, Lugui Qiu^1,2,*^, Yan Xu^1,2*^, Gang An^1,2,*^

**Contents:**

Figure S1-S6, Table S1-S5.


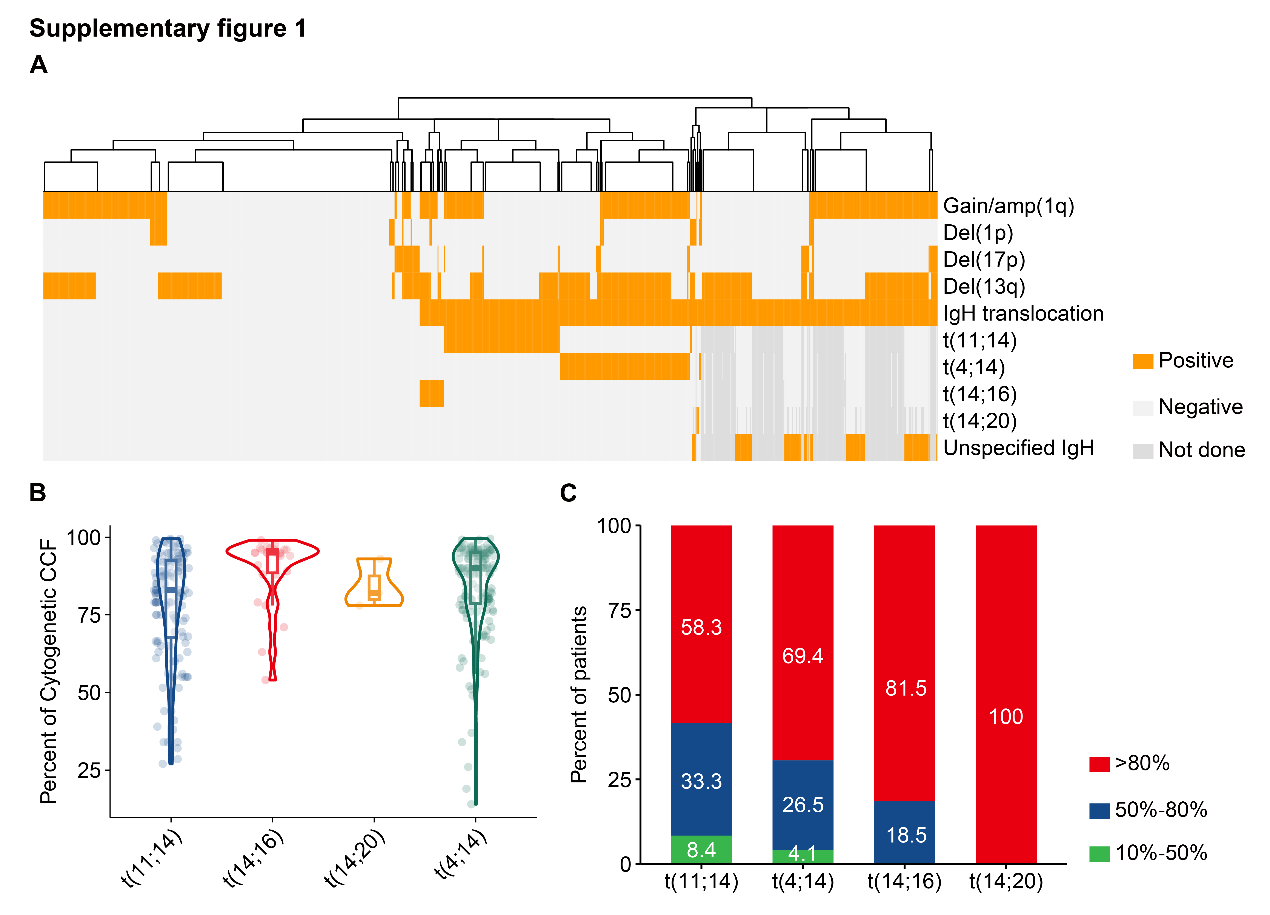


**Figure S1.**

**(A)** Heatmap showing the cytogenetic abnormalities detected by fluorescence *in situ* hybridization across 998 patients. **(B)** Volin plot of the cell fraction of t(11;14), t(14;16), t(14;20) and t(4;14). **(C)** Bar plot comparing the proportions of distribution of patients detected with different clonal sizes of IgH translocations.


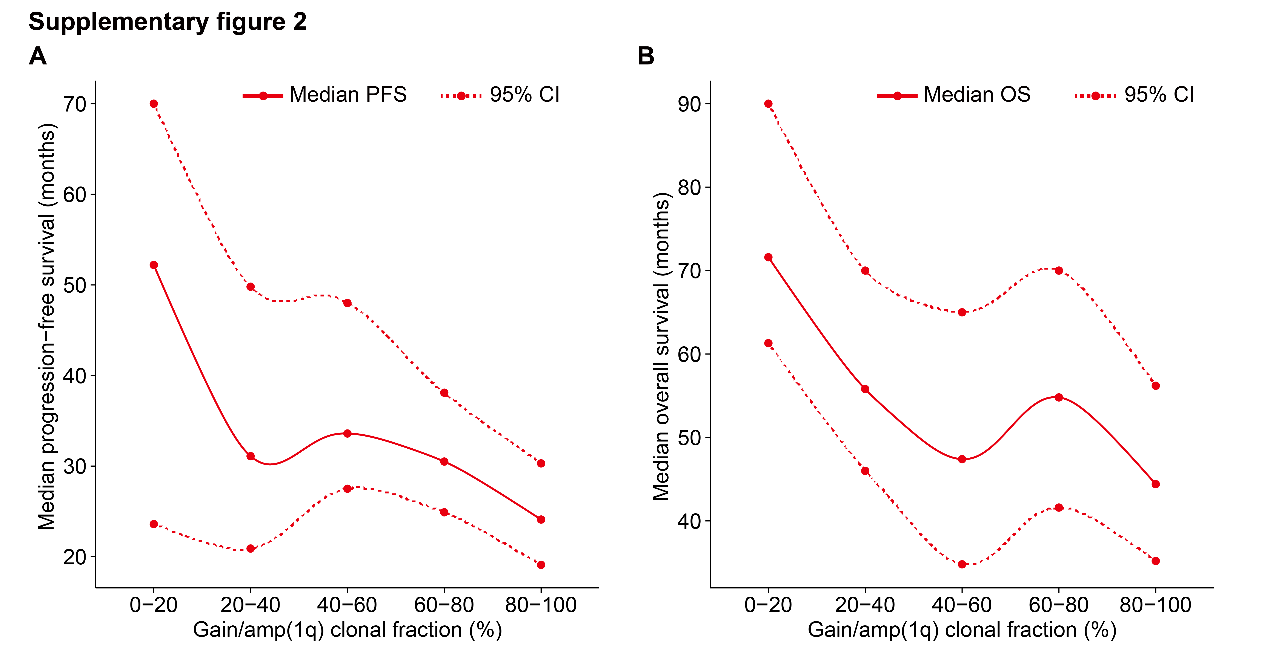


**Figure S2.**

**(A, B)** Line plots showing median PFS (A) and OS (B) for patients stratified by different clonal sizes of gain/amp(1q).


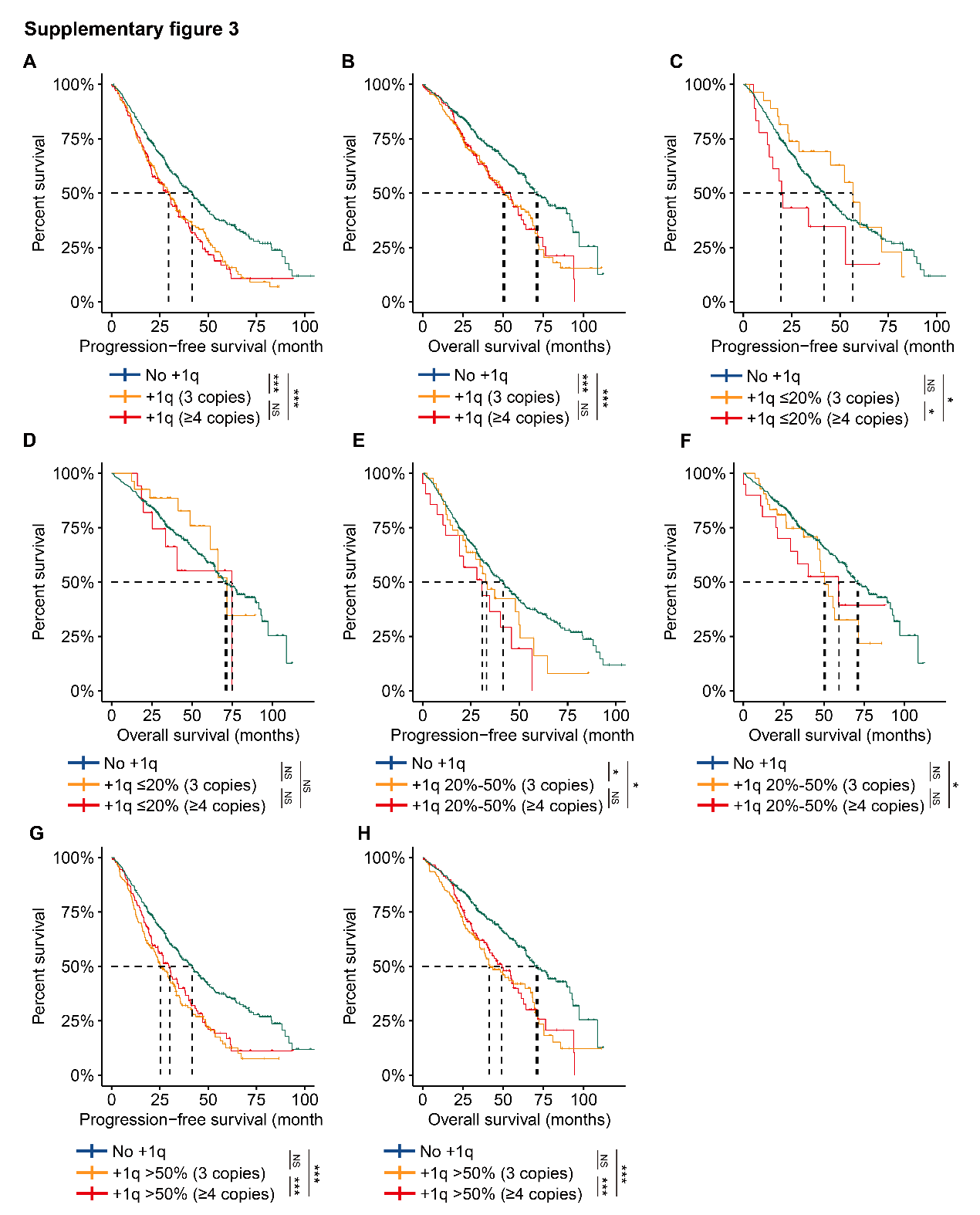


**Figure S3.**

**(A, B)** Kaplan-Meier analysis of PFS (A) and OS (B) by different copy number of gain/amp(1q). **(C-H)** Kaplan-Meier analysis of PFS (C) and OS (D) by different copy number of minor clone (C, D), subclonal clone (E-F) and dominant clone (G-H) of gain/amp(1q). NS, not significant, *P < 0.05, ***P < 0.001, by two-sided log-rank test.


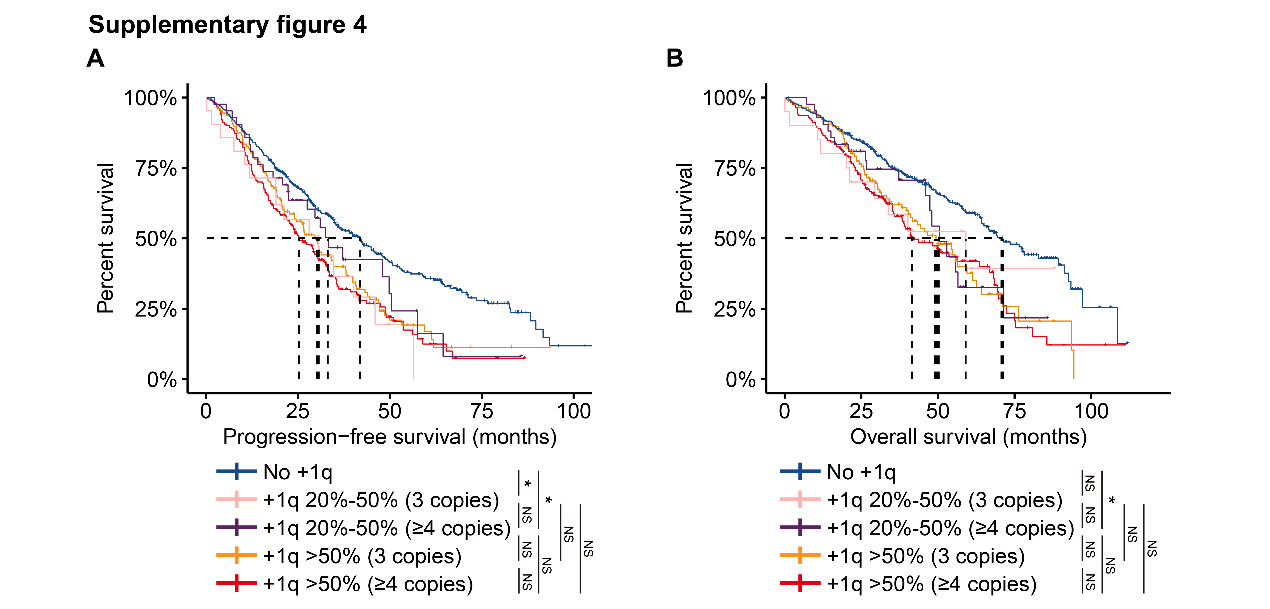


**Figure S4.**

**(A, B)** Kaplan-Meier analysis of PFS (A) and OS (B) by different clonal sizes and copy numbers in patients with gain/amp(1q).


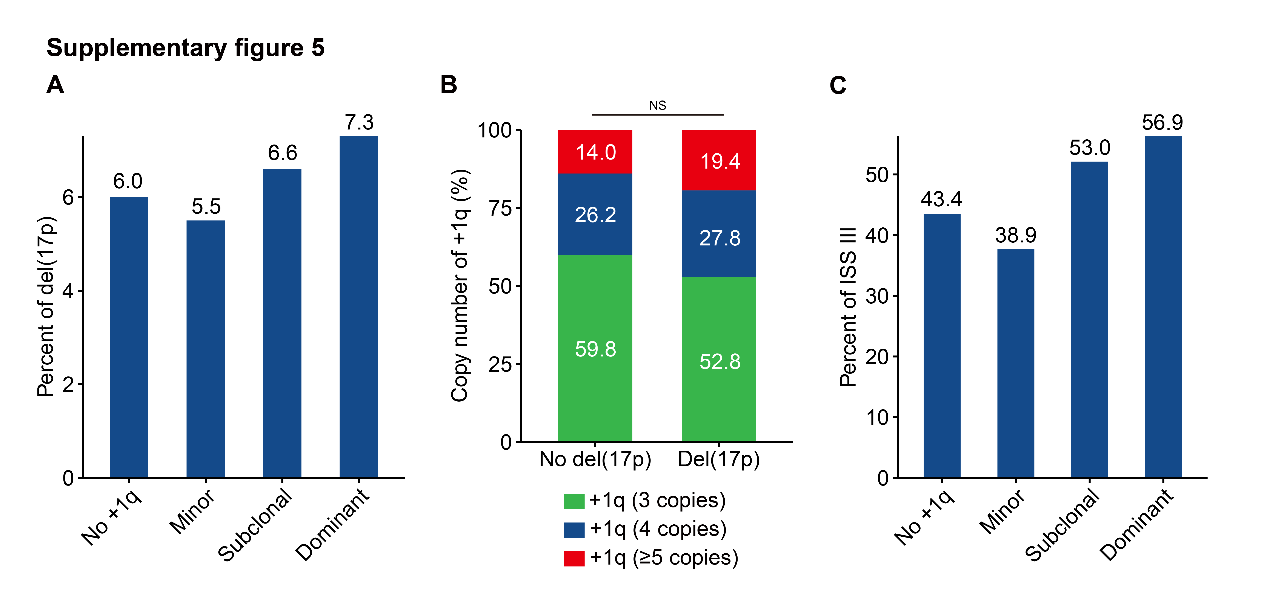


**Figure S5.**

**(A)** Percentage frequency of del(17p) associated with minor, subclonal, and clonal gain/amp(1q). **(B)** Bar plot comparing the proportions of distribution of patients detected with different copy numbers of 1q between patients with and without del(17p). NS, not significant, by 2-sided χ² test. **(C)** Percentage frequency of ISS stage III associated with minor, subclonal, and clonal gain/amp(1q).


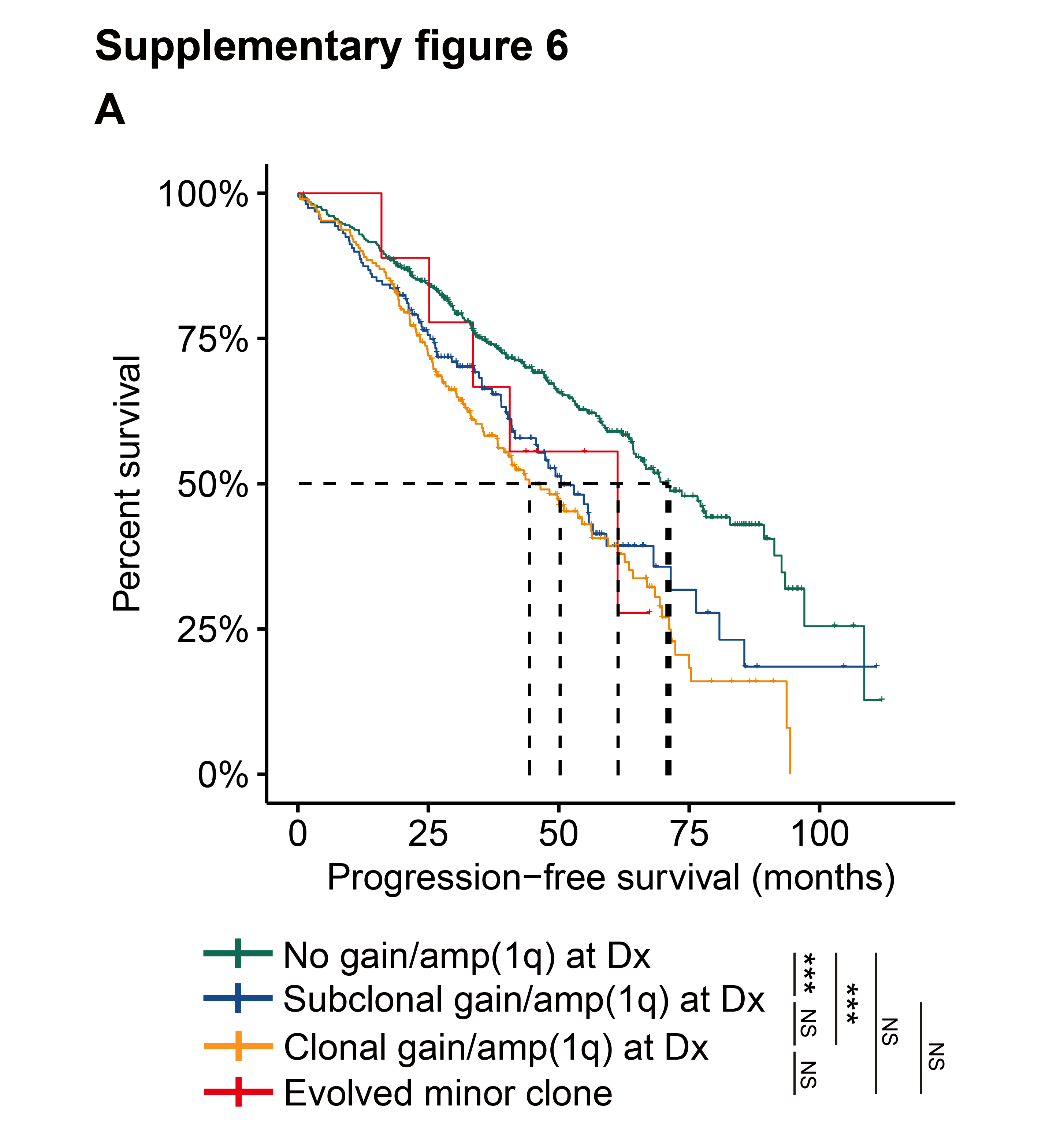


**Figure S6**

**(A)** Kaplan-Meier analysis of OS by different gain/amp(1q) clonal sizes at diagnosis and evolved minor clone between two time points. NS, not significant, ***P < 0.001, by two-sided log-rank test.

**Table S1. The cutoffs for gain/amp(1q) used in different centers.**

| **Group** | **Reference** | **Method** | **Cutoff value** |
| --- | --- | --- | --- |
| EMN, Europe | Haematologica. 2012;97(8):1272-1277. | NA | 10% for fusion, 20% for numerical abnormalities |
| IFM, Europe | J Clin Oncol. 2010;28(30):4630-4634.  J Clin Oncol. 2012;30(16):1949-1952.  Blood. 2002;99(6):2185-2191.  Blood. 2007;109(8):3489-3495.  J Clin Oncol. 2019;37(19):1657-1665. | MACS-FISH | 60% for del(17p), 30% for gain/amp(1q), 10.3% for t(11;14), 9.8% for t(4;14), 74% for del(13q) |
| University of Heidelberg, Germany | Haematologica. 2010;95(7):1150-1157.  Cancer. 2011;117(10):2136-2144.  Blood. 2012;119(4):940-948.  Haematologica. 2021;106(10):2754-2758. | MACS-FISH | 10% for gains, deletions, and translocations, 60%-70% for del(17p) |
| University of Arkansas, USA | Blood. 2000;96(4):1505-1511.  Blood. 2006;108(5):1724-1732.  Blood. 2008;112(10):4235-4246. | cig-FISH | 20% for del(17p), 20% for gain/amp(1q), 10% for del(17p) |
| Mayo Clinic, USA | Leukemia. 2001;15:981-986.  Cancer Res. 2002;62(3):715-720.  Blood. 2002;100(4):1417-1424.  Leukemia. 2006;20(11):2034-2040.  Blood Cancer Journal. 2019;9(32).  Blood Adv. 2019;3(13):1930-1938. | cig-FISH | 20% for gain/amp(1q), 10% for del(13q), 7% for del(17p), 25% for translocations |
| Mayo Clinic, USA | Blood Adv. 2020;4(15):3509-3519. | Unsorted | 3.5 for gain/amp(1q) |
| MD Anderson Cancer Center, USA | Blood Cancer Journal. 2024;14(1):4. | Unsorted | 7.9% for gain(1q), 0% for amp(1q), 4.7% for del(17p), 0% for t(4;14), 0% for t(14;16) |
| MD Anderson Cancer Center, USA | Biol Blood Marrow Transplant. 2016;22(12):2159-2164. | MACS-FISH | 6% for gain(1q), 4.4% for amp(1q) |
| MD Anderson Cancer Center, USA | Biol Blood Marrow Transplant. 2020;26(4):665-671. | Unsorted | 20% for gain/amp(1q) |
| Toronto General Hospital, Canada | J Clin Oncol. 2005;23(28):7069-7073.  Br J Haematol. 2006;134(6):613-615.  Br J Haematol. 2007;139(1):51-54.  Bone Marrow Transplant. 2010;45(1):117-121. | cig-FISH | 10% for gains, deletions, and translocations |
| National and Kapodistrian University of Athens, Greece | Am J Hematol. 2022;97(9):1142-1149. | CD138+ selected cells | 20% for gain/amp(1q) |
| Blood Diseases Hospital, China | Haematologica. 2014;99(2):353-359.  Clin Cancer Res. 2015;21(9):2148-2156.  Haematologica. 2023 Aug 3. | MACS-FISH | 10% for deletions and translocations, 20% for gain/amp(1q), 50% for del(17p) |
| First Hospital of Jilin University, China | Am J Hematol. 2023;98(2):251-263. | MACS-FISH | 5.5% for gain/amp(1q) and t(14;16), 20% for del(17p), 4.8% for del(13q), 7.3 for t(4;14), 6.3% for t(11;14), |
| Zhongshan Hospital, China | Leukemia & Lymphoma. 2020;61(10):2351-2364. | MACS-FISH | 5% for gain/amp(1q) |
|  | Cancer. 2023;129(7):1005-1016. |  |  |
| Beijing Chaoyang Hospital, China | Front Oncol. 2020;10:538126.  Cancer Med. 2020;9(21):7819-7829. | CD138+ selected cells | 10% for fusion, 20% for numerical abnormalities |
| The First Affiliated Hospital of Sun Yat-sen University, China | Oncologist. 2019;24(11):e1132-e1140. | CD138+ selected cells | 20% for gain/amp(1q) |
| Myeloma IX and Myeloma XI trials | Leukemia. 2018;32(1):102-110.  Lancet Oncol. 2019;20(1):57-73.  Haematologica. 2021;106(10):2754-2758. | CD138+ selected cells | 20% for gain/amp(1q) |
| Forte trial | Lancet Oncol. 2023;24(1):64-76. | CD138+ selected cells | 15% for translocations, 10% for deletions and gain(1q), 20% for amp(1q) |

Abbreviations: *cig-FISH* fluorescence in situ hybridization combined with cytoplasm immunoglobulin staining; *MACS-FISH* fluorescence in situ hybridization combined with magnetic activated cell sorting; *NA* not applicable.

**Table S2. Survival differences between MM patients with gain(1q) and amp(1q) in different studies.**

| **Reference** | **Patient resources** | **Cutoff value for +1q** | **Survival differences: Gain(1q) vs. Amp(1q)** |
| --- | --- | --- | --- |
| **Studies show no impact of the number of copies of 1q21 on the prognostic value** | | | |
| Haematologica. 2014. 99(2): 353-359. | Blood Diseases Hospital, China | 20% for gain/amp(1q) | PFS: 14 months vs. 10 months, P = 0.737;  OS: 24 months vs. 30 months, P = 0.382 |
| Leukemia & Lymphoma. 2020. 61(5): 1201-1210. | Blood Diseases Hospital, China | 20% for gain/amp(1q) | PFS: 23.2 months vs. 22.0 months, P = 0.593;  OS: 39.5 months vs. 40.9 months, P = 0.928 |
| Biol Blood Marrow Transplant. 2020. 26(4): 665-671. | MD Anderson Cancer Center, USA | 20% for gain/amp(1q) | PFS: 32.1 months vs. 20.0 months, P = 0.06;  OS: NR vs. NR, P = 0.84 |
| Am J Hematol. 2022. 97(9): 1142-1149. | National and Kapodistrian University of Athens, Greece | 20% for gain/amp(1q) | No prognostic impact on the number of copies of 1q21 either for PFS or OS |
| Blood Adv. 2020. 4(15): 3509-3519. | Mayo Clinic, USA | 3.5% for gain/amp(1q) | TTNT: 19.6 months vs. 14.4 months, P = 0.10;  OS: 4.9 years vs. 4.3 years, P = 0.21 |
| Cancer. 2023. 129(7): 1005-1016. | Zhongshan Hospital, China | 5% for gain/amp(1q) | No prognostic impact on the number of copies of 1q21 either for PFS or OS |
| Ther Adv Hematol. 2022. 13: 20406207221082043. | University of Hong Kong, Hong Kong | 20% for gain/amp(1q) | PFS: 39 months vs. 35 months, P = 0.853;  OS: 74 months vs. 61 months, P = 0.937 |
| Haematologica. 2021. 106(10): 2754-2758. | GMMG trials and Myeloma XI trial | GMMG trials: 10% for gain/amp(1q)  Myeloma XI trial: 20% for gain/amp(1q) | No prognostic impact on the number of copies of 1q21 either for PFS or OS |
| Leukemia. 2018. 32(1): 102-110. | Myeloma IX and Myeloma XI trials | 20% for gain/amp(1q) | PFS: HR = 0.91, P = 0.54;  OS: HR = 1.36, P = 0.09 |
| Cancer Med. 2020. 9(21): 7819-7829. | Beijing Chaoyang Hospital, China | 20% for gain/amp(1q) | PFS: 39 months vs. 34 months vs. 36 months, P = 0.404;  OS: 86 months vs. 61 months vs. 57 months, P = 0.554 |
| Oncologist. 2019. 24(11): e1132-e1140. | The First Affiliated Hospital of Sun Yat-sen University, China | 20% for gain/amp(1q) | PFS: 21.1 months vs. 30.0 months, P = 0.673;  OS: NR vs. 53.0 months, P =0.800 |
| Am J Cancer Res. 2021;11(9):4438-4454 | Clinic University Hospital Virgen de la Arrrixaca, Spain | 10% for gain/amp(1q) | 10-year PFS: 42.5% vs. 43.4%, P = 0.209;  10-year OS: 57.5% vs. 65.7%, P = 0.961 |
| J Clin Oncol. 2024: JCO2301277. | A large training cohort (N = 1933) | NA | PFS: P = 0.4493; OS: P = 0.2368 |
| **Studies show impact of the number of copies of 1q21 on the prognostic value** | | | |
| Blood Cancer J. 2019. 9(12): 94. | Emory University, USA | 20% for gain/amp(1q) | PFS: 55.9 months vs. 34.6 months, HR = 1.67 (0.89-3.12) |
| Blood Cancer J. 2019. 9(12): 94. | CoMMpass study | NA | PFS: P = 0.019; OS: P = 062 |
| Leukemia. 2019. 33(1): 159-170. | Myeloma Genome Project | NA | 18-months PFS: 71% vs. 60%, P = 0.06;  18-months OS: 88% vs. 73%, P = 0.08 |
| J Clin Oncol. 2015. 33(33): 3911-3920. | Myeloma XI trial | NA | PFS: HR = 1.8 (1.2-2.76), P = 0.004;  OS: HR = 2.7 (1.6-4.5), P < 0.001 |

Abbreviations: *NA* not applicable.

**Table S3. Baseline demographic and clinical characteristics of patients without gain/amp(1q) or with different clonal sizes of gain/amp(1q).**

| **Characteristic** | No +1q (N = 485) | Minor (N = 55) | Subclonal (N = 76) | Dominant (N = 382) | *P*-value |
| --- | --- | --- | --- | --- | --- |
| **Age, years, median (IQR)** | 59 (53-65) | 60.5 (48-67.5) | 59.5 (53-65) | 60 (51-67) | 0.929 |
| **Male** | 271 (54.7) | 29 (52.7) | 48 (63.2) | 209 (54.3) | 0.862 |
| **ISS stage, n (%)** |  |  |  |  | <0.01 |
| 1 | 99/446 (22.2) | 11/53 (20.8) | 11/73 (15.1) | 44/352 (12.5) |  |
| 2 | 153/446 (34.3) | 22/53 (41.5) | 24/73 (32.9) | 110/352 (31.3) |  |
| 3 | 194/446 (43.5) | 20/53 (37.7) | 38/73 (52.1) | 198/352 (56.3) |  |
| **R-ISS stage, n (%)** |  |  |  |  | <0.01 |
| 1 | 73/414 (17.6) | 8/49 (16.3) | 6/67 (9.0) | 22/301 (7.3) |  |
| 2 | 272/414 (65.7) | 28/49 (57.1) | 44/67 (65.7) | 185/301 (61.5) |  |
| 3 | 69/414 (16.7) | 13/49 (26.5) | 17/67 (25.4) | 94/301 (31.2) |  |
| **Hb, g/dL, median (IQR)** | 10.4 (8.6-12.5) | 10.2 (7.9-12.7) | 9.9 (7.9-12.4) | 9.0 (7.6-11.1) | <0.01 |
| **Platelets, ×10^9^/L, median (IQR)** | 199 (147-259) | 191 (119-244) | 216 (149-275) | 161 (115-213) | <0.01 |
| **Serum creatinine, umol/L, median (IQR)** | 80 (63-115) | 77 (54-104) | 78 (60-114) | 86 (69-127) | 0.857 |
| **LDH, units/L, median (IQR)** | 169 (137-208) | 188 (134-244) | 168 (140-247) | 170 (137-232) | 0.919 |
| **B2M, ug/mL, median (IQR)** | 4.2 (2.8-7.6) | 3.8 (2.8-5.6) | 4.4 (3.1-7.8) | 5.7 (3.9-9.9) | 0.190 |
| **High-risk cytogenetic, n (%)** |  |  |  |  |  |
| Any HRCA^a^ (n = 840) | 74/418 (17.7) | 10/46 (21.7) | 17/62 (27.4) | 132/314 (42.0) | <0.01 |
| IgH translocation | 237 (48.9) | 33 (60.0) | 50 (65.8) | 258 (67.5) | <0.01 |
| t(4;14) (n = 839) | 40/422 (9.5) | 7/45 (15.6) | 11/61 (18.0) | 89/311 (28.6) | <0.01 |
| t(11;14) (n = 831) | 80/415 (19.3) | 8/46 (17.4) | 14/60 (23.3) | 30/310 (9.7) | <0.01 |
| t(14;16) (n = 836) | 6/420 (1.4) | 1/45 (2.2) | 1/61 (1.6) | 19/310 (6.1) | <0.01 |
| t(14;20) (n = 809) | 2/409 (0.3) | 0/44 (0) | 0/61 (0) | 1/295 (0.5) | 0.907 |
| t(14;undefined)^b^ (n = 828) | 39/415 (9.4) | 7/45 (15.6) | 8/60 (13.3) | 45/308 (14.6) | 0.151 |
| del(17p) | 29 (6.0) | 3 (5.5) | 5 (6.6) | 28 (7.3) | 0.862 |
| del(13q) | 185 (38.1) | 22 (40.0) | 35 (46.1) | 235 (61.5) | <0.01 |
| del(1p) | 12 (2.5) | 3 (5.5) | 6 (7.9) | 30 (7.9) | <0.01 |
| **Treatment regimens, n/N (%)** |  |  |  |  | 0.173 |
| PI-containing induction | 264/348 (75.9) | 39/48 (81.3) | 50/61 (82.0) | 199/272 (73.2) |  |
| IMiD-containing induction | 51/348 (14.7) | 6/48 (12.5) | 2/61 (3.3) | 44/272 (16.2) |  |
| PI+IMiD-induction | 27/348 (7.8) | 2/48 (4.2) | 9/61 (14.8) | 25/272 (9.2) |  |
| Others | 6/348 (1.7) | 0/48 (0) | 0/61 (0) | 4/272 (1.5) |  |
| **First-line transplantation^c^, n/N (%)** | 112/326 (34.4) | 16/38 (42.1) | 15/58 (25.9) | 77/226 (34.1) | 0.416 |

^e^High-risk CA: presence of t (4;14), t(14;16), and/or del(17p).

^b^t(14; undefined): patients with an undefined abnormality of the 14q32 locus that did not correspond to one of the above three described common translocations.

^c^First-line transplantation was defined as transplantation within 12 months of first-line induction therapy.

Abbreviations: *IQR*, interquartile range; *ISS*, International Staging System; *R-ISS*, Revised International Staging System; *Hb*, hemoglobin; *LDH*, lactate dehydrogenase; *B2M*, β2-microglobulin; *HRCA* high-risk chromosome abnormality; *PI* proteasome inhibitor; *IMiD* immunomodulatory drug.

**Table S4. Longitudinal cytogenetic architecture studies in patients with minor clone of gain/amp(1q) at diagnosis (n = 13).**

| Patient | Sex | Age | Sampling 2, years | Time point | Del(17p), % | +1q, % | Copy number  of 1q21 | Del(13q), % | Del(1p) | t(11;14) | t(4;14) | t(14;16) | t(14; un)^a^ |
| --- | --- | --- | --- | --- | --- | --- | --- | --- | --- | --- | --- | --- | --- |
| 147 | M | 49 | 1.26 | Diagnosis | 24.5 | 6 | 4 | 58.5 | 92 | N | N | N | Y |
|  |  |  |  | Relapse | 44 | 24 | 5 | 14 | 16 | N | N | N | Y |
| 151 | M | 50 | 4.35 | Diagnosis | 1 | 17.5 | 3 | 23.5 | 1 | N | N | N | Y  Y |
|  |  |  |  | Relapse | 1 | 11 | 3 | 65 | 1 | N | N | N | Y |
| 254 | M | 45 | 1.97 | Diagnosis | 1 | 6.5 | 3 | 1 | 0 | N | N | N | N |
|  |  |  |  | Relapse | 2 | 48 | 3 | 2 | 1 | N | N | N | N |
| 281 | F | 49 | 1.11 | Diagnosis | 1 | 13 | 5 | 82 | 13 | Y | N | N | N |
|  |  |  |  | Relapse | 20 | 44 | 4 | 47 | 44 | Y | N | N | N |
| 489 | F | 65 | 0.53 | Diagnosis | 1 | 14 | 5 | 2.5 | 0.5 | N | N | N | N |
|  |  |  |  | Relapse | 2 | 53 | 5 | 4 | 0 | N | N | N | N |
| 836 | F | 58 | 4.41 | Diagnosis | 2.5 | 12 | 4 | 0.5 | 0 | N | N | N | Y  Y |
|  |  |  |  | Relapse | 2 | 1 | 2 | 3 | 0 | N | N | N | Y |
| 860 | F | 52 | 4.72 | Diagnosis | 0 | 15 | 3 | 2 | 0.5 | Y | N | N | N |
|  |  |  |  | Relapse | 0 | 52 | 3 | 4 | 0 | Y | N | N | N |
| 908 | M | 62 | 1.03 | Diagnosis | 2 | 15.5 | 4 | 8.5 | 0.5 | N | N | N | Y  Y |
|  |  |  |  | Relapse | 2.5 | 75.5 | 3 | 27 | 0 | N | N | N | Y |
| 928 | F | 63 | 0.67 | Diagnosis | 13 | 14 | 6 | 22 | 1 | N | Y  Y | N | N |
|  |  |  |  | Relapse | 38 | 87 | 3 | 57 | 0 | N | Y | N | N |
| 933 | M | 51 | 2.39 | Diagnosis | 2.5 | 18 | 3 | 4.5 | 0 | N | N | N | N |
|  |  |  |  | Relapse | 2.5 | 14 | 4 | 4.5 | 0 | N | N | N | N |
| 942 | F | 63 | 0.47 | Diagnosis | 2 | 15 | 4 | 3 | 1 | N | N | N | N |
|  |  |  |  | Relapse | 1.5 | 0 | 2 | 2 | 0 | N | N | N | N |
| 947 | M | 53 | 1.70 | Diagnosis | 2 | 12 | 5 | 4.5 | 0 | N | N | N | N |
|  |  |  |  | Relapse | 1 | 97 | 5 | 2.5 | 0 | N | N | N | N |
| 997 | F | 62 | 0.45 | Diagnosis | 0 | 17.5 | 4 | 0 | 14 | N | N | N | Y |
|  |  |  |  | Relapse | 0 | 97 | 3 | 0 | 84 | N | N | N | Y |

^a^t(14; undefined): patients with an undefined abnormality of the 14q32 locus that did not correspond to one of the above three described common translocations.

**Table S5. Overview of patients’ treatment and response characteristics in patients with minor clone of gain/amp(1q) at diagnosis (n = 13).**

| Patient ID | Age | ISS | R-ISS | Induction | End of Induction Response | ASCT | Maintenance | PFS | PFS (Months) | OS | OS (Months) |
| --- | --- | --- | --- | --- | --- | --- | --- | --- | --- | --- | --- |
| 147 | 49 | 3 | 3 | BCD | CR | Y | TD | Y | 15.1 | Y | 40.6 |
| 151 | 50 | 1 | NA | BCD | CR | N | R | Y | 52.2 | Y | 66.0 |
| 154 | 45 | 3 | 2 | BD | CR | N | MPT | Y | 23.6 | Y | 61.3 |
| 281 | 49 | 1 | 1 | BCD | VGPR | Y | TD | Y | 13.4 | Y | 33.6 |
| 489 | 65 | 2 | 2 | BCD | PD | N | NA | Y | 6.3 | N | 43.7 |
| 836 | 58 | 3 | 3 | BCD | VGPR | N | ID | Y | 52.9 | Y | 74.6 |
| 860 | 52 | 3 | 2 | BCD | PR | Y | TD | Y | 56.7 | N | 67.4 |
| 908 | 62 | 3 | 2 | BCD | VGPR | Y | R | Y | 12.4 | N | 45.7 |
| 928 | 63 | 3 | 3 | VRD | PR | N | RD | Y | 8.1 | Y | 25.2 |
| 933 | 51 | 1 | 2 | VTD | VGPR | N | ND | Y | 28.7 | Y | 48.7 |
| 942 | 63 | 2 | 2 | D-VMP | PR | N | NA | Y | 5.6 | N | 24.8 |
| 947 | 53 | 3 | 2 | PAD | PR | N | NA | Y | 20.4 | N | 54.9 |
| 997 | 62 | 3 | 2 | BD | PD | N | NA | Y | 5.4 | Y | 16.0 |

Abbreviations: *ISS*, International Staging System; *R-ISS*, Revised International Staging System; *PFS* progression-free survival; *OS* overall survival; *BCD* bortezomib, cyclophosphamide and dexamethasone; *BD* bortezomib and dexamethasone; *VRD* bortezomib, lenalidomide and dexamethasone; *VTD* bortezomib, thalidomide and dexamethasone; *D-VMP* daratumumab, bortezomib, melphalan and prednisone; *PAD* bortezomib, doxorubicin and dexamethasone; *CR* complete response; *VGPR* very good partial response; *PR* partial response; *PD* progressive disease; *TD* thalidomide and dexamethasone; *R* lenalidomide; *MPT* melphalan, prednisone and thalidomide; *ID* ixazomib and dexamethasone; *RD* lenalidomide and dexamethasone; *NA* not applicable; *ND* not done.
